# Tumor and serum gamma-glutamyl transpeptidase, new prognostic and molecular interpretation of an old biomarker in gastric cancer

**DOI:** 10.18632/oncotarget.15609

**Published:** 2017-02-22

**Authors:** Qinchuan Wang, Xiang Shu, Yong Dong, Jichun Zhou, Rongyue Teng, Jianguo Shen, Yongxia Chen, Mingjun Dong, Wenjun Zhang, Yasheng Huang, Shuduo Xie, Qun Wei, Wenhe Zhao, Wenjun Chen, Xiaoming Yuan, Xu Qi, Linbo Wang

**Affiliations:** ^1^ Department of Surgical Oncology, Affiliated Sir Runrun Shaw Hospital, Zhejiang University School of Medicine, Hangzhou, China; ^2^ Department of Oncology, Affiliated Sir Runrun Shaw Hospital, Zhejiang University School of Medicine, Hangzhou, China; ^3^ Zhejiang Academy of Medical Science, Zhejiang University School of Medicine, Hangzhou, China; ^4^ Division of Epidemiology, Department of Medicine, Vanderbilt-Ingram Cancer Center, Vanderbilt University School of Medicine, Nashville, TN, USA; ^5^ Department of Unrology, Hangzhou Chinese Medicine Hospital, Hangzhou, China

**Keywords:** gamma-glutamyl transpeptidase, gastric cancer, prognosis, serum, chemotherapy

## Abstract

**Background:**

Gastric Cancer is one of the most lethal malignancies worldwide. Gamma-glutamyl transpeptidase (GGT) is an enzyme mainly involved in cellular glutathione homeostasis. We aim to explore the clinical value of GGT in gastric cancer.

**Results:**

Among 322 patients enrolled, 65/82 patients were determined as GGT positive in serum/tumor, respectively. High tumor GGT expression is significantly associated with lymph node metastasis, histological subtype, and Her2 expression. Kaplan-Meier curve shows that high tumor GGT patients have shorter overall survival (*P _log-rank_*=0.001) and progress-free survival (*P _log-rank_* =0.001). Patients with both high tumor and serum GGT have the poorest prognosis. The multivariable Cox analysis shows that the hazard ratio of overall survival for high tumor GGT is 1.69 (95% CI 1.19-2.37). High serum GGT is a poor prognostic factor in adjuvant chemotherapy hazard ratio=2.18, 95%CI (1.15-4.47). These findings were further validated in six online datasets. Gene Sets Enrichment Analysis showed that GGT promotes cancer progression through EMT, KRAS, SRC and PKCA pathways.

**Methods:**

Tumor GGT and serum GGT levels were evaluated with immuno-histochemistry staining and enzymatic assay, respectively. Kaplan-Meier curve and Cox regression model were used to test the association between GGT and gastric cancer prognosis. Independent datasets from Gene Expression Omnibus and Gene Sets Enrichment Analysis were applied to validate the findings and explore the potential mechanisms.

**Conclusion:**

Both tumor GGT and serum GGT are poor prognostic factors in gastric cancer. Patients with high tumor and serum GGT levels require more intense treatment and follow-up.

## INTRODUCTION

Gastric cancer was the world's third leading cause of cancer deaths, which was estimated to be responsible for nearly 723,000 deaths in 2016 [1]. In China, there are estimated 679,000 new cases and 498,000 deaths from gastric cancer in 2015 [2]. Despite the recent advances in treatment, recurrence rates are still high and the 5-year survival rate for all stages remains low at 25% [3].

Currently, the classification of gastric cancer subtypes is mainly based on histology, for instance, Lauren classification and World Health Organization classification [4]. Till now, only 2 biomarkers are implemented in the clinic, which are based on Her2 protein overexpression and/or amplification of its gene ERBB2. The two biomarkers can be used to guide the use of trastuzumab in gastric cancer treatment. However, Her2 positive expression was only found in 20-30% of gastric cancer patients, which limits its usage in other patients [5]. A project was conducted by The Cancer Genome Atlas (TCGA) to develop molecular classifiers of gastric adenocarcinoma, which subdivided the cancer into 4 subtypes based on its genomic alterations [6]. Also, using immunohistochemistry staining and in situ hybridization, a 14-maker classifier was suggested by Lauwers, et al [7]. However, the clinical values of these classifiers await further verifications, and more biomarkers and targets are urgently needed for the detection and treatment of gastric cancer.

Gamma-Glutamyl transpeptidase (GGT) is a cell surface, N-terminal nucleophile hydrolase involved in cellular glutathione homeostasis. As glutathione is the main water-soluble antioxidant in the cell, GGT is usually activated under oxidative stress like hepatic injury caused by alcohol, drugs, and hepatitis, which is widely applied in the monitoring of liver function [8]. GGT has been demonstrated as an independent risk factor for many chronic diseases [9–12]. A Korean study of 1.6 million individuals identified that serum GGT levels are significantly associated with gastric cancer risk in men (HR=1.04, 95% CI=1.03–1.05), but not in women [13]. Besides its association with cancer risk, tumor GGT expression was also reported correlated with cancer progression and drug resistance in several cancer sites [14, 15]. For instance, it is believed that increased expression of GGT in cancer cells is accompanied by increased invasiveness in melanoma [16], and cisplatin resistance due to excess glutathione production in Hela cells [17]. Moreover, in peptic ulcer disease and gastric cancer, *H.pylori* derived GGT was proposed as an important factor causing oxidative DNA damage in the development of disease [18, 19]. High tumor GGT mRNA expression was found associated with poor prognosis in multiple cancers (http://www.prognoscan.org/, Supplementary Figure 1). Serum GGT level was reported as partially independent of tumor GGT expression [14, 20]. However, whether and how the serum GGT and tumor GGT expression interact in cancer patients, and their clinical values in gastric cancer, are not clear.

In the current study, to explore the prognostic role of GGT in gastric cancer, we measured GGT levels in tumor tissues and sera collected from 322 gastric adenocarcinoma patients. We further validated our results and explored the potential mechanisms in the cohorts of gastric cancer patients from public databases.

## RESULTS

### High GGT expressions in tumor are associated with gastric cancer subtypes and lymph node metastasis

**Figure 1:**
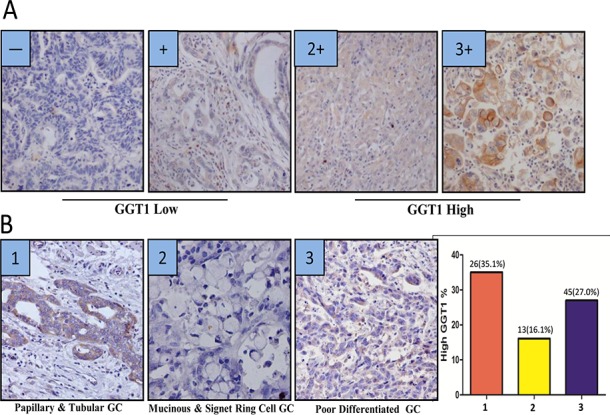
GGT expression is different among histological subtypes of gastric cancer (A) The scoring criteria of cytoplasmic GGT in gastric cancer, 0 refers negative, + refers weak positive, 2+ refers positive, 3+ refers strong positive 0 and + are considered as GGT low, while 2+ and 3+ are considered as GGT high. **(B)** Representative image of GGT staining in gastric cancer subtypes. Image 1 is one of papillary & tubular gastric adenocarcinoma, image 2 is mucinous & Signet Ring Cell Adenocarcinoma, image 3 is poor differentiated adenocarcinoma. A summary graph was also plotted in right panel, showing the positive percentage of each subtype (1= papillary & tubular gastric adenocarcinoma: 26/74, 2= mucinous & Signet Ring Cell Adenocarcinoma: 13/81, 3= poor differentiated adenocarcinoma: 45/167).

IHC staining of GGT was performed on 322 cases of gastric cancer. 82 (25.4%) out of 322 patients were identified as GGT positive (IHC 2+ and 3+). For baseline levels of serum GGT (sGGT), 239 patients’ data were available. Among them, 24 pg/ml was defined as the cut-off point based on a previous publication [13], and 65 (27.2%) out of 239 patients were defined as sGGT high. A demographic analysis was applied on the associations among GGT, sGGT and clinicopathological variables (Table 1). Tumor GGT was significantly associated with sex, lymph node involvement, histological subtypes, tumor nodular formation and Her2 expression (*p*<0.05 each), and marginally correlated with distant metastasis (*p*=0.09) and tumor grade (*p*=0.13); whereas sGGT showed marginal correlation with histological subtype (*p*=0.11) and Her2 expression (*p*=0.12). Representative pictures of each subtype were shown in Figure 1B. Tumor GGT is preferentially expressed in papillary and tubular adenocarcinoma (26/74), whereas its expression is relatively lower in mucinous & signet ring cell cancer (13/81) and poor-differentiated adenocarcinoma (45/167).

**Table 1:** Correlations between clinic-pathological features of GC patients with tumor and serum GGT expression

		GGT			sGGT		
		Cases	%of GGT(+)a	*P* value	Cases	%of sGGT (+)a	*P* value
Age							
	<60	160	36 (22.5)		109	30(27.5)	
	>=60	162	46 (28.4)	0.22	130	35(26.9)	0.92
Sex							
	Male	217	64 (29.5)		168	48(27.4)	
	Female	105	18 (17.1)	*0.02	71	17(26.6)	0.89
Location of tumor^b^							
	Proximal^c^	59	14 (23.7)		40	10(25.0)	
	Body^d^	67	25 (37.3)		53	18(34.0)	
	Distal^e^	175	38 (21.7)		135	31(23.0)	
	Whole^f^	11	3 (27.3)	0.11	7	3(42.1)	0.36
TNM stages^b^							
	Stage I & II	138	31 (22.5)		126	31(24.6)	
	Stage III &IV	178	49 (27.5)	0.3	111	32(28.8)	0.46
Invasion Depth^b^							
	T1&T2	80	18 (22.5)		85	20(23.5)	
	T3&T4	236	62 (26.3)	0.5	150	42(28.0)	0.45
Lymph node^b^							
	Negative	86	14 (16.3)		86	21(24.4)	
	Positive	224	65 (29.0)	*0.017	148	40(27.0)	0.66
Distant Metastasis							
	No	281	67 (23.8)		212	55(25.9)	
	Yes	41	15 (36.6)	**0.09**	27	10(37.0)	0.24
Tumor grade							
	Low (G1)	42	16 (38.1)		46	10(21.7)	
	Moderate (G2)	78	20 (25.6)		57	13(22.8)	
	High (G3 & G4)	202	46 (22.8)	0.13	136	42(30.9)	0.33
Histological type							
	Papillary & Tubular	74	26(35.1)		69	20(29.0)	
	Mucinous & Signet Ring Cell	81	13(16.1)		59	10(17.0)	
	Poor differentiated	167	45(27.0)	*0.02	110	34(30.9)	0.12
Vascular Invasion							
	Yes	24	14 (58.3)		36	59(26.7)	
	No	274	114 (41.6)	0.11	196	6(33.3)	0.55
Tumor Nodular Formation							
	Yes	48	18(23.0)		30	5(16.7)	
	No	261	60(37.5)	*0.04	202	56(27.7)	0.18
Lesion Size (Largest dimension)							
	<5 cm	126	29 (23.0)		128	34(26.6)	
	>=5cm	177	46 (26.0)	0.55	105	27(25.7)	0.88
Her2 expression							
	Negative	94	35 (37.2)		136	38(27.9)	
	Positive	185	38 (20.5)	*0.003	43	7(16.3)	**0.11**
Ki67 Index							
	Low	85	20 (23.5)		110	10(23.3)	
	High	172	47 (27.3)	0.51	43	28(25.5)	0.78

NOTE: All samples were collected from Zhejiang University. All information about TNM stage (tumor) were based on the pathological report of surgical specimens according to NCCN gastric cancer guideline v 2011.2

^a^ GGT(+) means immunohistochemical staining score 2+ and 3+, and sGGT(+) means serum GGT level >24 pg/ml.

^b^ All missing cases were appropriately coded as “missing value”

^c^ Proximal stomach includes: Cardia, GEJ, Esophagus lower, fundus.

^d^ Body stomach includes: lesser curve, greater curve, stomach overlapping, body.

^e^ Distal stomach includes: Gastric antrum, pylorus

^f^ Whole stomach indicates: linitis plastica

* indicates p<0.01, statistical significance.

### High GGT expressions in tumor and serum predict poor outcome in gastric cancer patients

**Figure 2:**
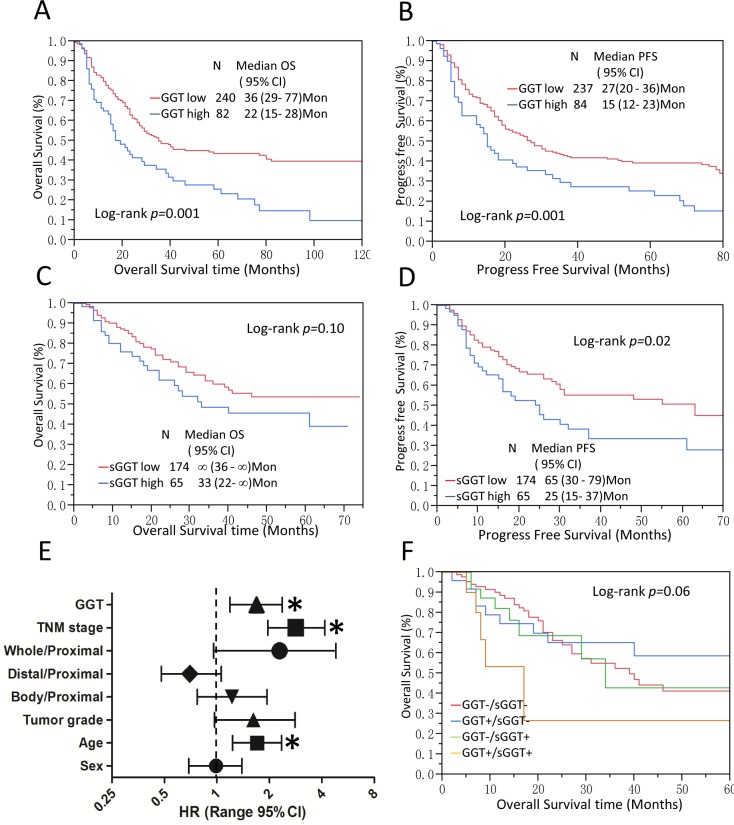
GGT is a poor prognostic factor in gastric cancer **(A-D)** Kaplan-Meier Analysis was conducted to calculate the impact of tumor GGT **(A-B)** and serum GGT **(C-D)** on OS and PFS of gastric cancer patients. **(E)** Multivariate Cox analysis for tumor GGT levels and OS are shown in (E). * P < 0.05, tumor location: Proximal tumor were set as reference. **(F)** tumor GGT and sGGT significantly impacted survival in gastric cancer patients. Median OS for GGT-/sGGT-(red), GGT+/sGGT- (blue), GGT-/sGGT+ (green), and GGT+/sGGT+ (yellow) were 39, 27, 26, and 17 months, respectively. GGT+/sGGT+ subgroup showed the poorest survival compared to other subgroups (log-rank p=0.06).

In our study, high GGT expression in tumor was strikingly correlated with poor OS and PFS in gastric cancer patients (*p*=0.001 each) (Figure 2A–2B), high GGT group showed massively reduced overall and progression-free survival against low GGT group (median OS time: 36 months *vs*. 22 months; median PFS time: 27 months *vs*. 15 months). Interestingly, sGGT was also found to be significantly associated with cancer recurrence, high sGGT group demonstrated shorter PFS than patients with low sGGT levels (median PFS 65 months *vs*. 25 months, *p*=0.02) (Figure 2D). However, sGGT only showed a marginal impact on overall survival (*p*=0.10) (Figure 2C).

To minimize potential confounding, the multivariable COX proportional hazard analysis was employed. Age, sex, tumor location, tumor grade and TNM stage were adjusted in the model. As we illustrated in Figure 2E, tumor GGT (HR=1.69, 95% CI=1.19-2.37), TNM stage (HR=2.83, 95% CI=1.96-4.17), tumor grade (HR=1.62, 95% CI=0.97-2.81) and age at diagnosis (HR=1.70, 95% CI=1.23-2.35) were significant prognostic factors of OS (detail data in Supplementary Table 2). However, sGGT showed little impact on OS (HR=1.04 95% CI=0.61-1.73) (Supplementary Table 3). Further stratified analysis showed high tumor GGT is a prognostic factor for patients who had distal gastric adenocarcinoma, advanced stage (stage III & IV), lower tumor grade (G1 & G2), positive Her2 expression and surgery only, respectively (Table 2). Propensity score matching was also applied to further rule out confounding factors (Supplementary Table 4&5).

**Table 2:** Stratification analysis for tumor GGT expression and overall survival of GC patients

	GC Patients (n=322)		
	No. of Cases	HR (95% CI)	Adjusted HR (95% CI)
**TNM stage**			
Stage0, I & II	138	1.93 (0.98-3.60)	1.88 (0.94-3.56)
Stage III & IV	178	**1.63 (1.11-2.36)**	**1.63 (1.10-2.39)**
**Tumor location^a^**			
Proximal	59	1.26 (0.55-2.60)	1.19 (0.51-2.59)
Body	67	1.21 (0.63-2,27)	1.48 (0.76-2.85)
Distal	175	**2.45 (1.51-3.88)**	**2.40 (1.46-3.86)**
**Tumor Grade**			
Low	202	**1.91 (1.27-2.80)**	**1.98 (1.31-2.96)**
Moderate	78	**2.20 (1.11-4.24)**	**2.09 (1.02-2.96)**
High	42	1.72 (0.59-5.04)	2.40 (0.74-7.91)
**Histological subtype**			
Papillary & Tubular	77	**2.16 (1.10-4.33)**	**2.20 (1.10-4.50)**
Mucinous & Signet	84	**3.81 (1.53-8.25)**	**4.0 (1.58-8.87)**
Ring Cell			
Undifferentiated	161	**1.64 (1.05-2.50)**	**1.65 (1.04-2.56)**
**Her2**			
Negative	185	1.56 (0.95-2.45)	1.64 (0.99-2.59)
Positive	94	**2.41 (1.39-4.18)**	**2.42 (1.35-4.35)**
**Chemotherapy**			
No	169	**2.17 (1.39-3.30)**	**2.14 (1.38-3.27)**
Yes	153	1.54 (0.92-2.47)	1.52 (0.91-2.48)

NOTE: Multivariate COX proportional hazard analysis was conducted to evaluate HR of GGT high versus low, tumor GGT low group was used as reference. The HRs were adjusted by sex and age at diagnosis.

*Statistical significant on COX proportional hazard analysis, *p*<0.05

^a^,11 cases of “whole” (linitis plastica) are not analyzed due to insufficient numbers.

To investigate potential interaction between sGGT and tumor GGT on GC outcomes, Kaplan-Meier analysis was conducted. Interestingly, GGT(+)/sGGT(+)subgroup showed the poorest OS (n=12, median OS=17 months) among all subgroups, followed by GGT(+)/sGGT(−)(n=27, median OS=34 months), GGT(−)/sGGT(−)(n=88, median OS=39 months), and GGT(−)/sGGT(+)(n=26, median OS not applicable) (Figure 2F).

### GGT is associated with chemo-resistance in gastric cancer patients

**Figure 3:**
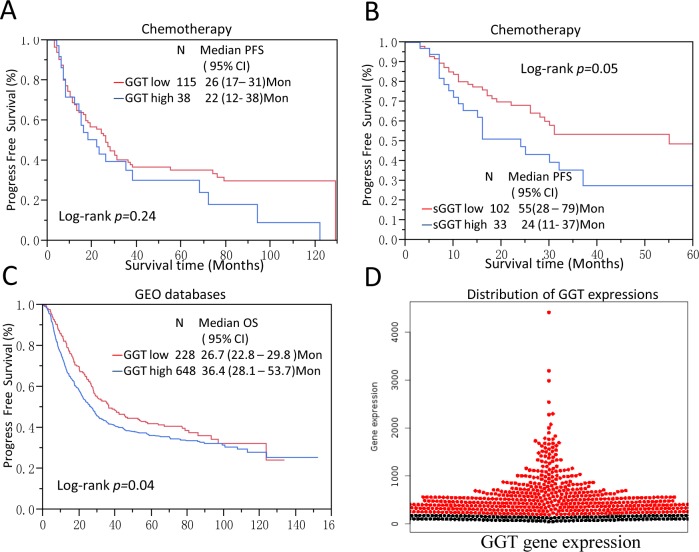
GGT levels are correlated with chemo-resistance of gastric cancer and validation on public databases **(A, B)** Serum GGT level are marginally associated PFS (p=0.05) in gastric cancer patients who received 5-Fu and platinum based chemotherapy, but tumor GGT expression is not significantly correlated with PFS (p=0.24); **(C, D)** The result was further validated in a pooled analysis of six GEO datasets which were assembled inwww.kmplot.com. In validation dataset there are 876 patients’ information and gene expression data available. Patients with high GGT expression showed significant poor overall survival compare to low GGT expression patients. *All the probes are normalized and dichotomized according to previous publication [45].

It has been suggested that GGT may cause cisplatin resistance in HeLa cells model [17]. To further explore the role of GGT in gastric cancer chemotherapy resistance, Kaplan-Meier survival analysis and COX proportional hazard model were applied to patients who received adjuvant chemotherapy in SRRSH set. In 153 gastric cancer patients who received 5-FU and Platinum-based chemotherapy, high tumor GGT subgroup showed no significant association (*p*=0.24) with PFS (Figure 3A). Interestingly, high sGGT significantly associated with reduced PFS of GC patients (*p*=0.05) (Figure 3B). A multivariable COX proportional hazard analysis showed that high sGGT is a significant predictor of tumor relapse in patients who had chemotherapy (HR=2.18, 95%CI 1.15-4.47), but not in surgery-alone patients (Table 3).

**Table 3:** Multivariate COX proportional hazard analysis for PFS of GCs

Factors	Surgery alone	Chemotherapy
	HR (95% CI)	HR (95% CI)
sGGT		
low	Reference	Reference
high	0.71(0.39-1.35)	**2.18(1.15-4.47)†**
Location		
Proximal	Reference	Reference
Body	1.53(0.63-3.73)	0.98(0.46-2.10)
Distal	0.72(0.37-1.50)	0.57(0.33-1.05)
Whole*	N/A	N/A
TNM stage		
Stage I&II	Reference	Reference
StageIII&IV	1.68(0.86-3.52)	1.42(0.86-2.39)
Tumor Grade		
Low	Reference	Reference
High	1.23(0.63-2.38)	0.84(0.45-1.54)
Age (per unit)		
<60	Reference	Reference
>=60	0.88(0.51-1.56)	1.05(0.65-1.71)
Gender		
Female	Reference	Reference
Male	1.34(0.73-2.37)	0.85(0.47-1.47)

Note: Multivariate COX proportional hazard analysis was conducted to evaluate HR of sGGT1 for PFS of GCs. *Patients in the whole group is not sufficient to do the analysis.

† Statistical significance, *p*<0.05.

To further validate our results, 6 cohorts of gastric patients from GEO were aggregated and normalized. (GEO accession number: GSE14210, GSE15459, GSE51105, GSE62254, GSE22377, GSE29272). All basic information of these datasets was listed in Supplementary Table 1. The mRNA expression of tumor GGT was normalized and dichotomized at KMplot.com [21]. Kaplan-Meier analysis showed high GGT mRNA expression is a poor prognostic factor in gastric patients (*P _Logrank_*=0.04) (Figure 3C–3D). While in the one of the cohort with chemotherapy (GSE14210), high GGT expression showed as a marginal factor of Progress-free survival (PFS) (Supplementary Figure 2).

**Figure 4:**
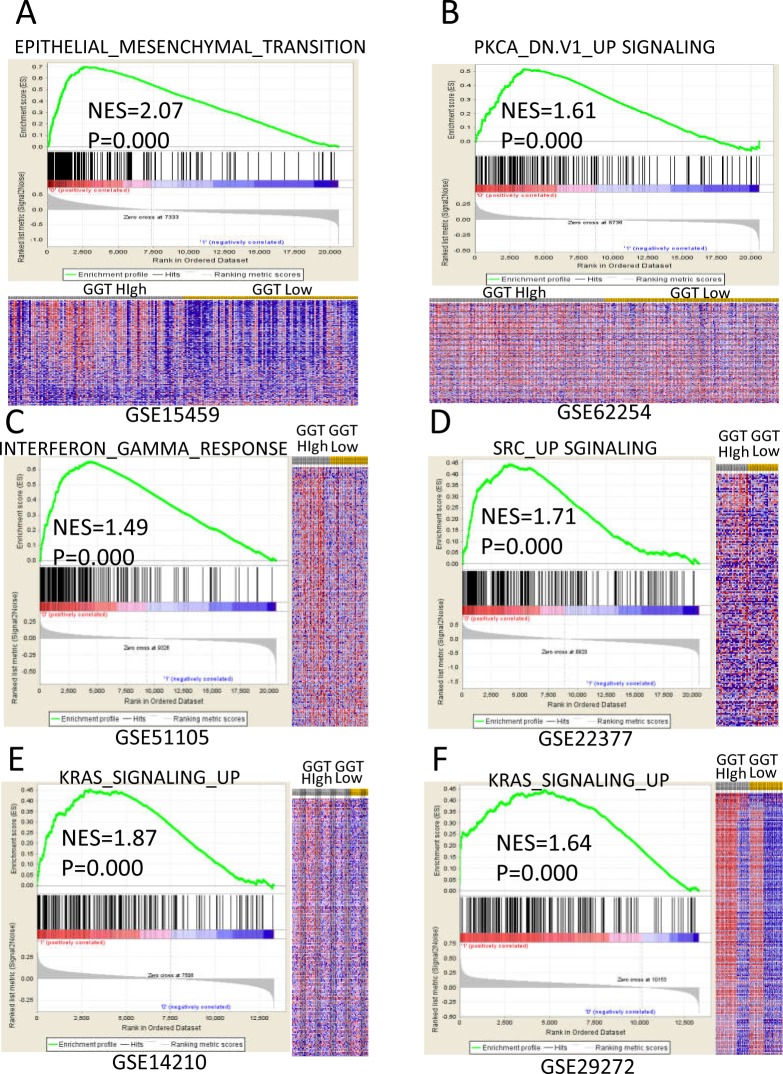
Enriched gene signatures of high GGT group are associated with proliferation and metastasis of gastric cancer NES (Normalized Enrichment Score) represents score for the gene-set enrichment analyses. The ranked list metric was generated by calculating the signal-to-noise ratio, which is based on the difference of means scaled according to the standard deviation. The signal-to-noise ratio determine the distinction of a gene expression for each phenotype, which makes the gene acts as a “class marker”. The detailed information of computational method is list in the website of The Broad Institute Gene Set Enrichment Analysis website (www.broad.mit.edu/gsea). The heat maps show the enrichment of genes in the gene sets. Rows represent each gene, and columns are individual samples. Each cell in the matrix represents the expression level of a gene in an individual sample. Red indicates a high level of expression, and green indicates a low level of expression. In each dataset, the most up-regulated enriched gene set in GGT-high (annotated as high in the figure) group was picked and listed as following: **(A)** Epithelial-Mesenchymal Transition (EMT) signature in GSE15459 dataset; **(B)** PKCA signaling in GSE62254 dataset; **(C)** Interferon Gamma Response signature in GSE51105 dataset; **(D)** SRC signaling in GSE22377 dataset; **(E&F)** KRAS signalingGSE14210 and GSE29272 datasets.

Further GSEA analysis was applied on six datasets. All samples were re-stratified as GGT high or GGT low according to median values of each dataset. Hallmark gene sets were selected in the analysis. Genes were significantly enriched in Epithelial-Mesenchymal Transition (EMT) signature (Dataset: GSE15459, NES=2.07, FDR<25%), PKCA signaling (Dataset: GSE62254, NES=1.61, FDR<25%), Interferon-gamma Response signature (Dataset: GSE51105, NES=1.49, FDR<25%) and KRAS signaling (Dataset: GSE14210 & GSE29272, NES=1.87 & 1.64, respectively, FDR<25%) for GGT high expression GC patients (Figure 4A through 4F). These findings indicate that GGT could promote cancer proliferation and metastasis through EMT, KRAS, SRC and PKCA signaling pathways.

## DISCUSSION

In this study, we investigated the role of tumor and serum GGT in gastric cancer prognosis and potential mechanisms. We reported that tumor GGT expressions are associated with sex, lymph node metastasis, histological subtype, tumor nodular formation and Her2 expression (each of *p*<0.05, respectively). High tumor GGT expression is a poor prognostic factor in GC, whereas high sGGT level is closely associated with disease recurrence. High tumor GGT expression and sGGT level predict poor outcome in GC patients. Moreover, high sGGT level is demonstrated as a resistant factor of 5-FU and platinum-based chemotherapy (p=0.05). Lastly, the findings were further validated in six datasets from GEO databases. GSEA analysis shows that EMT, KRAS, SRC and PKCA pathways are possible downstream signaling pathways of GGT in gastric carcinogenesis. To the best of our knowledge, this is the first study on GGT's clinical value and possible mechanisms in gastric cancer.

Our study provides evidence that tumor GGT expression is associated with gastric cancer lymph node metastasis and tumor nodular formation. As an anti-oxidative enzyme, GGT plays an important role in the cancer cell under stress [8]. It has been reported that GGT elevation is accompanied by an increased invasive behavior in melanoma and breast cancer, which is in accordance with our results [16, 22]. Our GSEA analysis also indicates that GGT is significantly correlated with EMT, KRAS, SRC and PKCA pathways, which are closely associated with cancer metastasis and proliferation [23, 24]. This may partially explain the mechanisms of invasive phenotype of high GGT group of GC patients.

GGT was reported as an important factor of chemo-resistance through various mechanisms [25]. Firstly, GGT could provide GSH and cysteine to cancer cells by cleaving extracellular GSH, which enable cancer cells resisting to the pro-oxidant chemo-agents [25]. For example, increased intracellular GSH and cysteine could form adducts with platinum, and reduce DNA toxicity of platinum to cancer cells [17, 26, 27]. Also, GGT-dependent pro-oxidant can induce redox modulation and the binding of NF-kB and AP-1 to DNA, which could exert proliferative and anti-apoptotic signals in cells [28–30], thereby increasing resistance of cells to the chemo-agents. This explains our results of high sGGT level relapse earlier in the chemotherapy group than surgery only group. Secondly, potential downstream signal pathways of GGT we found could contribute to the resistance. For example, EMT was recently reported as a key factor of chemo-resistance in lung cancer [31], so is the KRAS signaling in germ-cell tumors [32]. Therefore, our findings indicated that GGT could contribute to chemo-resistance by increasing intracellular GSH and downstream signal pathways.

Cancer-derived GGT in circulation has been described as a poor prognostic factor in several types of neoplasms, like renal cell carcinoma and hepatocellular carcinoma [12, 33]. In our study, elevated sGGT is a predictive factor for the recurrence in gastric cancer. Moreover, patients with high tumor GGT and sGGT have the poorest survival in our study. This indicates that tumor GGT level may has a joint effect with sGGT. Notably, sGGT is not correlated with tumor GGT expression (*p*=0.36, data not shown) in our study. This may cause by liver secretion or peripheral clearance of GGT. However, a large cohort of patients (N=283,438) from Austria suggested that elevated sGGT significantly increases cancer risk, regardless of liver diseases [34]. Also, we excluded the GC patients with liver dysfunction in our study. Therefore, based on our findings, tumor GGT and sGGT are predictive of poor outcome in gastric cancer, but sGGT may also affect by other factors.

Our findings are novel. First, serum GGT level was identified as a predictor of tumor relapse for the first time. We carefully considered and handled potential confounders, such as alcohol usage and liver dysfunctions. Secondly, we identified both the serum and tumor level of GGT expression in our cohort and find the joint effect between them. We also validated our results in several independent public datasets. Despite these strengths, we acknowledge several limitations of our study: First, this is a retrospective study in a single center, further validation is still required. Secondly, the evaluation methods of tumor GGT expression are different between our study (Immunohistochemistry) and publicly available GC cohorts (mRNA microarray). As several studies reported, protein staining and mRNA microarray could reach moderate correlation [35, 36]. However, post-transcription modifications, such as epigenetic changes, could affect the result [37]. Thirdly, the mechanisms between tumor and serum GGT interactions are not characterized in this study. We only explore the clinical values of tumor and serum GGT in gastric cancer patients, and further analyzed the possible mechanisms.

In summary, we demonstrated that both tumor and serum GGT levels are poor prognostic factors in gastric cancer patients. Tumor GGT expression and serum GGT has a joint effect on the poor outcome. EMT, KRAS, SRC and PKCA pathways may be the key signaling pathways in the GGT signaling in gastric cancer.

## MATERIALS AND METHODS

### Ethic statement

The protocol of this study was reviewed and approved by the Institutional Review Board (IRB) of Affiliated Sir Run Run Shaw Hospital (SRRSH), Zhejiang University. Written informed consent was obtained from all the patients enrolled in this study.

### Patients

We enrolled 472 Gastric Cancer patients with informed consent who were treated at the Department of Surgical Oncology in SRRSH between 1995 and 2011. The inclusion criteria were as follows: 1. Gastric adenocarcinoma with confirmed pathology diagnosis; 2. Received R0 resection and N2 lymphadenectomy; 3. Informed consent. The exclusion criteria were: 1. Patients with liver dysfunction, like hepatitis, alcohol abuse, etc.; 2. Non-adenocarcinoma or multiple cancers; 3. Lack of tissue sample; 4. Failure to obtain informed consent; 5. Fail to contact the patients after surgery. Finally, 322 patients were available for analysis. All patients are Han Chinese. Among these patients, 153 of 322 patients had post-surgery adjuvant chemotherapy. The combination chemotherapy regimens included folinic acid, 5-fluorouracil and oxaliplatin (FOLFOX6: 73 Cases); epirubicin, oxaliplatin and Xeloda (EOX: 12 cases); epirubicin, mitomycin and 5-fluorouracil (FEM: 9 cases); epirubicin, oxaliplatin and 5-fluorouracil (EOF: 38 cases); mitomycin C and 5-fluorouracil (4 cases) and others (oral S-1/x, docetaxel-based and other protocols; 17 cases). All patients were followed annually until January 2012 or loss of follow-up. The clinicopathological information of each patient was updated annually. The TNM stage data for the participants were obtained from the clinical and pathological diagnoses and determined according to the NCCN guidelines for GC (Version 2, 2015). The human tissue samples examined in this study were obtained from surgery and stored at room temperature after formalin-fixed and paraffin embedded. Correlation analysis suggested storage time did not significantly affect genes expression (*p*>0.05) [38].

### Microarray data sets

A total of 6 published microarray datasets were assembled and normalized according to a recently published paper [21], which is available atwww.kmplot.com: Rozen (GSE15459) [39], Green (GSE14210) [40], Förster (GSE22377) [41], Taylor (GSE29272) [42], Busuttil (GSE51105) [43], Loboda (GSE62254) [44]. Also, all the datasets’ annotations were downloaded from GEO (http://www.ncbi.nlm.nih.gov/geo/) (details summarized in Supplementary Table 1). The probes of GGT (208284_x_at, 211417_x_at, 209919_x_at, 215603_x_at, 207131_x_at) were normalized and blasted, which are 100% similarity to sequence of >gi|572153073|ref|NM_013421.2|.

For survival analysis, all data were dichotomized into GGT-low and GGT-high using R script according to the method in a previous publication [45].

### Study design

This is a population based outcome study in gastric cancer (Supplementary Figure 3). The sample size was calculated with nQuery Advisor 6.01 (Statistical Solutions Ltd, Saugus, MA, USA) software. Based on this, we deemed 300 participants to reach a 95% study power (two-side α=0.05). All demographic and clinic-pathological data were carefully collected through chart review and reassembled into a detailed database. All gastric cancer patients were periodically followed up for survival and disease recurrence. The overall survival (OS) period was calculated as the time from initial surgery to the date the patient was last seen or until Jan 2012. The progress-free survival (PFS) was defined as the time from initial surgery until tumor recurrence, including local relapse and metastasis.

### Gene set enrichment analysis (GSEA)

The detailed GSEA protocol was downloaded from the Broad Institute Gene Set Enrichment Analysis website (www.broad.mit.edu/gsea) [46]. The GSEA software v2.2.2 was run in JAVA 7.0 platform. The dataset (.gct) and phenotype label (.cls) files were created and loaded into GSEA software. The gene sets were downloaded from Board Institute website. The number of permutations was set to 1000, and the phenotype label was GGT_high versus GGT_low. The ranked-list metric was generated by calculating the signal-to-noise ratio, which is based on the difference of means scaled according to the standard deviation.

### Baseline serum GGT detection

Baseline serum GGT levels were determined with routine clinical biochemistry when patients were admitted. Total cholesterol, fasting glucose, alanine aminotransferase (ALT) and aspartate aminotransferase (AST) were also tested. Fasting blood samples were collected on the morning of admission before patients received any examination or treatment. Quality control of procedures was in accordance with the Westgard rules of Laboratory Quality Control Standards [47].

### Semi-quantitative immunohistochemistry

Immunohistochemistry (IHC) was applied to determine the expression levels of GGT on formalin-fixed paraffin-embedded (FFPE) human tissue samples. To normalize the reaction conditions, all FFPE tissue samples were reassembled into multiple tissue arrays as we previous reported [38].

Briefly, after deparaffinization, the endogenous peroxidase activity was blocked with 3% hydrogen peroxide (H_2_O_2_). The array slides were later incubated with normal goat serum for 20 minutes and then applied with primary antibody for 20 minutes at room temperature. After 7 minutes of H_2_O_2_ treatment, the array slides were incubated with horseradish peroxidase-labeled polymer conjugated diaminobenzidine (0.05 g of 3, 3-diaminobenzidine and 100 mL of 30% H_2_O_2_ in 100 mL of PBS) for 5 and 10 minutes, respectively. Each slide was then counterstained with hematoxylin (DAKO). PBS was used as a negative control. The accuracy of IHC was validated by quantitative RT-PCR (qRT-PCR) on two parallel samples. Antibody against GGT (1:200, Catalog #:ab55138), Ki67 (1:100, Clone: B56) and Her2 (1:200, A0485) were purchased from Abcam (Cambridge, MA), BD Bioscience (San Jose, CA) and DAKO (Denmark).

During the quantification of staining, to reduce the reader bias, we employed an automated imaging system to obtain digital images of the stained sections for subsequent quantitative analyses. Each sample was evaluated by two independent investigators in a double-blind manner. Cytoplasmic GGT, nuclear Ki67, and membranous Her2 were semi-quantified following our previous method [38, 48].

### Statistical analysis

All demographic data, clinic-pathological information, and IHC results were coded and entered into a GC database. Double data entry and logic checks were performed. The missing cases were labeled with the appropriate “missing” code. Kaplan–Meier analysis and Cox proportional hazard model were applied for the OS and PFS analyses. JMP 8.0 Software (SAS Institute, Cary, NC, USA) and GraphPad Prism 5.0 (GraphPad Software, Inc, La Jolla, CA, USA) were used for statistical analysis and survival curve plots. Propensity score matching was conducted with Stata13 (College Station, TX).

## SUPPLEMENTARY MATERIALS FIGURES AND TABLES





## Abbreviations

GC: Gastric Cancer
GGT: gamma-glutamyl transpeptidase
FFPE: formalin-fixed paraffin-embedded
HR: Hazard ratio
GEO: Gene Expression Omnibus
GSEA: Gene Sets Enrichment Analysis
EMT: Epithelial-Mesenchymal Transition
TCGA: The Cancer Genome Atlas
SRRSH: Sir Run Run Shaw Hospital
