# Supplementary material for: Tumor and serum gamma-glutamyl transpeptidase, new prognostic and molecular interpretation of an old biomarker in gastric cancer
Source: Oncotarget. 2017 Feb 22;8(22):36171–84. doi: 10.18632/oncotarget.15609 (PMC5482647; doi:10.18632/oncotarget.15609)
Supplement: Supplementary file 1 [file oncotarget-08-36171-s001.pdf]

Tumor and serum gamma-glutamyl transpeptidase, new prognostic and molecular interpretation of an old biomarker in gastric cancer

SUPPLEMENTARY FIGURES AND TABLES

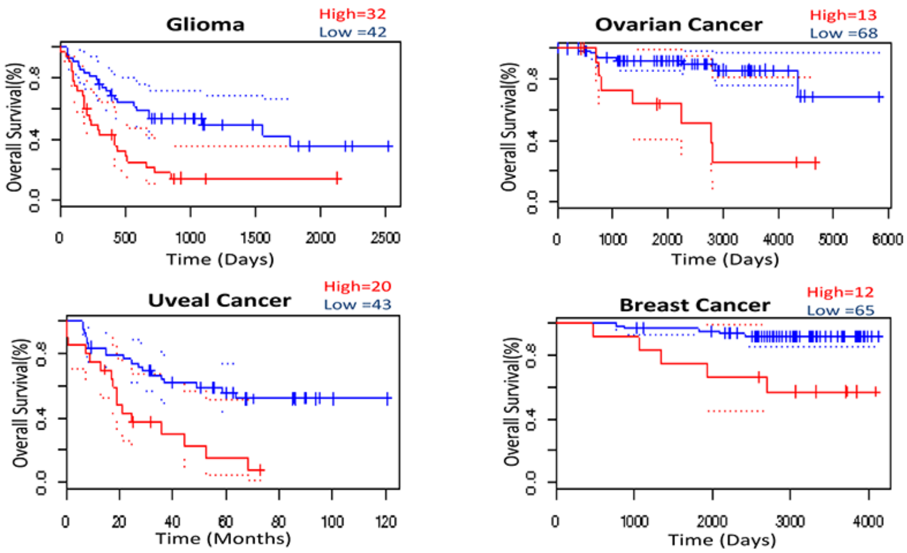

Supplementary Figure 1: High expression of GGT indicates poor prognosis in multiple cancer sites. Studies in glioma (N=74), ovarian cancer (N=81), uveal cancer (N=63) and breast cancer (N=77) indicate high GGT expression in tumor prognosticate poor outcomes. All data were assembled and analyzed in http://www.prognoscan.org/.

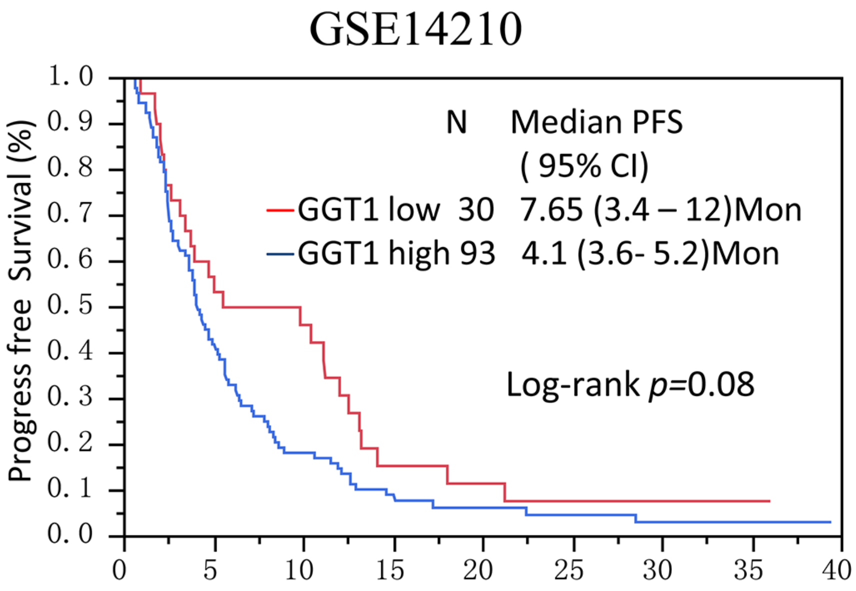

Supplementary Figure 2: In dataset GSE14210 from GEO database, low GGT expression patients showed marginal significance in progress free survival over high GGT expression counterparts in 123 metastatic gastric cancer patients who received cisplatin and 5-Fu combination chemotherapy (*p*=0.08).

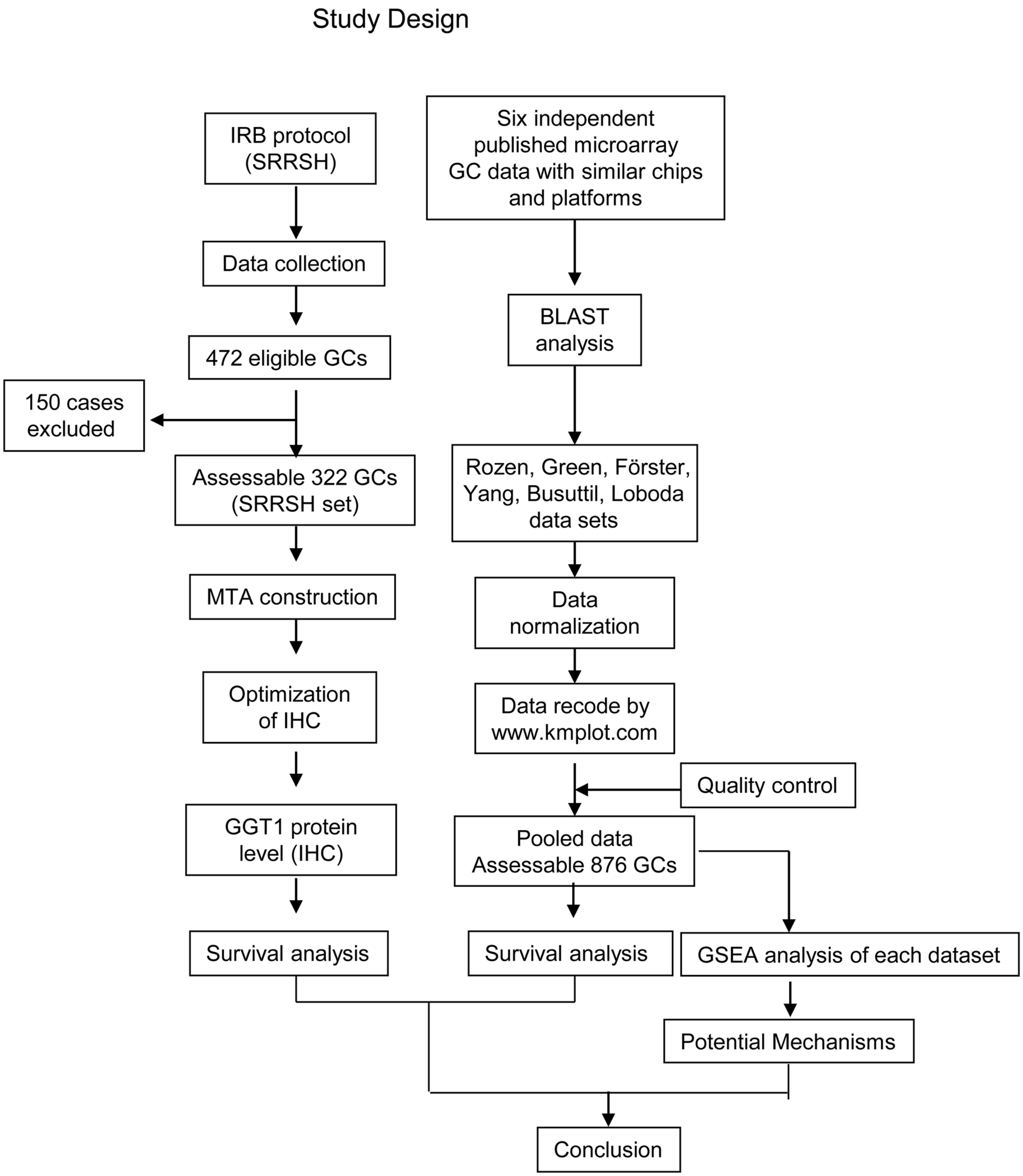

Supplementary Figure 3: The study design of the outcome study was listed as a flow chart.

Supplementary Table 1: Overall review of published microarray data sets

See Supplementary File 1

Supplementary Table 2: Multivariate COX proportional hazard analysis for OS of GCs

Factors	HR (95% CI)
GGT	
Low	Reference
High	**1.69(1.19-2.37)†**
Location	
Proximal	Reference
Body	1.22(0.77-1.94)
Distal	0.70(0.48-1.06)
Whole	2.29(0.96-4.83)
TNM stage	
Stage I&II	Reference
Stage III&IV	**2.83(1.96-4.17)†**
Tumor Grade	
Low	Reference
High	**1.62(0.97-2.81)†**
Age	
<60	Reference
>=60	**1.70(1.23-2.35)**
Gender	
Female	Reference
Male	0.99(0.69-1.39)

Note: Multivariate COX proportional hazard analysis was conducted to evaluate HR of GGT for overall survival of GCs.
† Statistical significance, $p<0.05$.

Supplementary Table 3: Multivariate COX proportional hazard analysis for OS of GCs (Serum GGT)

Factors	HR (95% CI)
sGGT	
low	Reference
high	1.04(0.61-1.73)
Location	
Proximal	Reference
Body	1.59(0.72-3.71)
Distal	1.24(0.64-2.64)
Whole	**4.98(1.61-14.3)†**
TNM stage	
Stage I&II	Reference
StageIII&IV	**4.19(2.47-7.40)†**
Tumor Grade	
Low	Reference
High	1.18(0.83-1.73)
Age	
<60	Reference
>=60	1.47(0.91-2.40)
Gender	
Female	Reference
Male	1.20(0.68-2.03)

Note: Multivariate COX proportional hazard analysis was conducted to evaluate HR of sGGT for overall survival of GCs.
† Statistical significance, $p<0.05$.

Supplementary Table 4: COX proportional hazard analysis for OS of GCs

Factors	HR (95% CI)
GGT	
Low	Reference
High	**1.93(1.33-2,77)**

Note: COX proportional hazard analysis was conducted to evaluate HR of GGT for overall survival of GCs. The cases were matched using propensity score matching based on 11 covariates (age, sex, TNM stage, tumor grade, tumor location, histological subtypes, tumor size, vascular invasion, tumor nodular formation, Her2 expression and Ki67 expression).
† Statistical significance, $p<0.05$.

Supplementary Table 5: COX proportional hazard analysis for PFS of GCs

Factors	HR (95% CI)
GGT	
Low	Reference
High	**1.74(1.25-2.43)**

Note: COX proportional hazard analysis was conducted to evaluate HR of GGT for progress free survival of GCs. The cases were matched using propensity score matching based on 11 covariates (age, sex, TNM stage, tumor grade, tumor location, histological subtypes, tumor size, vascular invasion, tumor nodular formation, Her2 expression and Ki67 expression).

† Statistical significance, p<0.05.
