# Supplementary material for: Tumor and serum gamma-glutamyl transpeptidase, new prognostic and molecular interpretation of an old biomarker in gastric cancer
Source: Oncotarget. 2017 Feb 22;8(22):36171–84. doi: 10.18632/oncotarget.15609 (PMC5482647; doi:10.18632/oncotarget.15609)
Supplement: Supplementary file 2 [file oncotarget-08-36171-s002.docx]

| **Supplementary Table 1: Overall review of published microarray data sets** | | | | | | |
| --- | --- | --- | --- | --- | --- | --- |
| **Data set** | **Rozen** | **Green** | **Förster** | **Yang** | **Busuttil** | **Loboda** |
| No. of patients | 200 | 123 | 43 | 268 | 94 | 300 |
| Assessable cases* | 200 | 123 | 43 | 126 | 93 | 283 |
| Date of study | 2009 | 2011 | 2011 | 2013 | 2014 | 2015 |
| Microarray | AffimetrixHG-U133 | AffimetrixHG-U133 Plus2.0 | Affymetrix U133 Plus 2.0 Array | Affymetrix U133A Array | Affymetrix U133 Plus 2.0 Array | Affymetrix U133 Plus 2.0 Array |
|  |  |  |  |  |  |  |
| Accession No. | GSE15459 | GSE14210 | GSE22377 | GSE29272 | GSE51105 | GSE62254 |
| GGT1 probes |  |  |  |  |  |  |
| 208284_x_at | Y | Y | Y | Y | Y | Y |
| 211417_x_at | Y | Y | Y | Y | Y | Y |
| 209919_x_at | Y | Y | Y | Y | Y | Y |
| 215603_x_at | Y | Y | Y | Y | Y | Y |
| 207131_x_at | Y | Y | Y | Y | Y | Y |
| Age at diagnosis | 73 | N/A | N/A | N/A | N/A | N/A |
|  | (23-92) |  |  |  |  |  |
| Hist. type | Y | N/A | Y | N/A | N/A | N/A |
| Tumor grade | N/A | N/A | N/A | N/A | N/A | N/A |
| Tumor size | N/A | N/A | N/A | N/A | N/A | N/A |
| Lymph node | N/A | N/A | N/A | N/A | N/A | N/A |
| Metastasis | N/A | N/A | N/A | N/A | N/A | N/A |
| AJCC stage | Y | Y | Y | Y | Y | Y |
| HER2 status | N/A | N/A | N/A | N/A | N/A | N/A |
| MKI67 status | N/A | N/A | N/A | N/A | N/A | N/A |
| Chemotherapy | N/A | Y | N/A | N/A | N/A | N/A |
| Radiotherapy | N/A | Y | N/A | N/A | N/A | N/A |
| OS months† | 19 | 9.2 | 35.9 | 25.7 | 29 | 59 |
| (Range) | 0-157.3 | 1-39.4 | 8-152.2 | 2.5-93.7 | 0.7-129.8 | 1-105.7 |
| PFS months‡ | N/A | 4.4 | N/A | N/A | N/A | N/A |
| (Range) |  | 0.6-39.4 |  |  |  |  |
